# Presentation and physical therapy management using a neuroplasticity approach for patients with hypermobility-related upper cervical instability: a brief report

**DOI:** 10.3389/fneur.2024.1459115

**Published:** 2024-11-08

**Authors:** Susan Chalela, Leslie N. Russek

**Affiliations:** ^1^The Chalela Physical Therapy Institute for EDS/CCI and Cervical Instabilities, Charleston, SC, United States; ^2^Health and Rehabilitative Sciences Department, Medical University of South Carolina, Charleston, SC, United States; ^3^Physical Therapy Department, Clarkson University, Potsdam, NY, United States; ^4^Physical Therapy Department, St. Lawrence Health System, Potsdam, NY, United States

**Keywords:** hypermobility, cervical instability, physical therapy, neuroplasticity, Ehlers-Danlos Syndrome, proprioception, biofeedback, upper cervical instability

## Abstract

**Background:**

Upper cervical instability (UCI) is a potentially disabling complication of the connective tissue disorders hypermobile Ehlers-Danlos Syndrome and Hypermobility Spectrum Disorders (hEDS/HSD). UCI can impact various neurological structures, including the brainstem, spinal cord, cranial nerves, and blood supply to and from the brain, resulting in complex neurological signs and symptoms in this population. The current study was an observational study applying recent expert consensus recommendations for physical therapy assessment and management of patients with UCI associated with hEDS/HSD.

**Methods:**

This was a retrospective observational study describing how the clinical decision-making model was used to screen, examine, and treat three patients with highly irritable hEDS/HSD-related UCI, resulting in complex neurological presentation. The treatment used a neuroplasticity approach, including proprioception and motor control training emphasizing patient education and biofeedback. Outcome measures tracked progress.

**Results:**

All patients started with significant disability associated with UCI. One patient returned to full function with intermittent flares that he was able to manage. The second patient continued to have mild-moderate irritability but returned to parenting responsibilities and full-time work. The third patient required cervical fusion and remained disabled but was better able to minimize flares. The number of initial red and yellow flags was associated with the final outcomes, suggesting that the decision-making model might be useful for predicting patient prognosis.

**Conclusion:**

This brief report applies recent recommendations for safely evaluating and managing hypermobility-related UCI and provides a first step in experimental studies to test both the assessment and physical therapy treatment approaches.

## 1 Introduction

**Figure 1:**
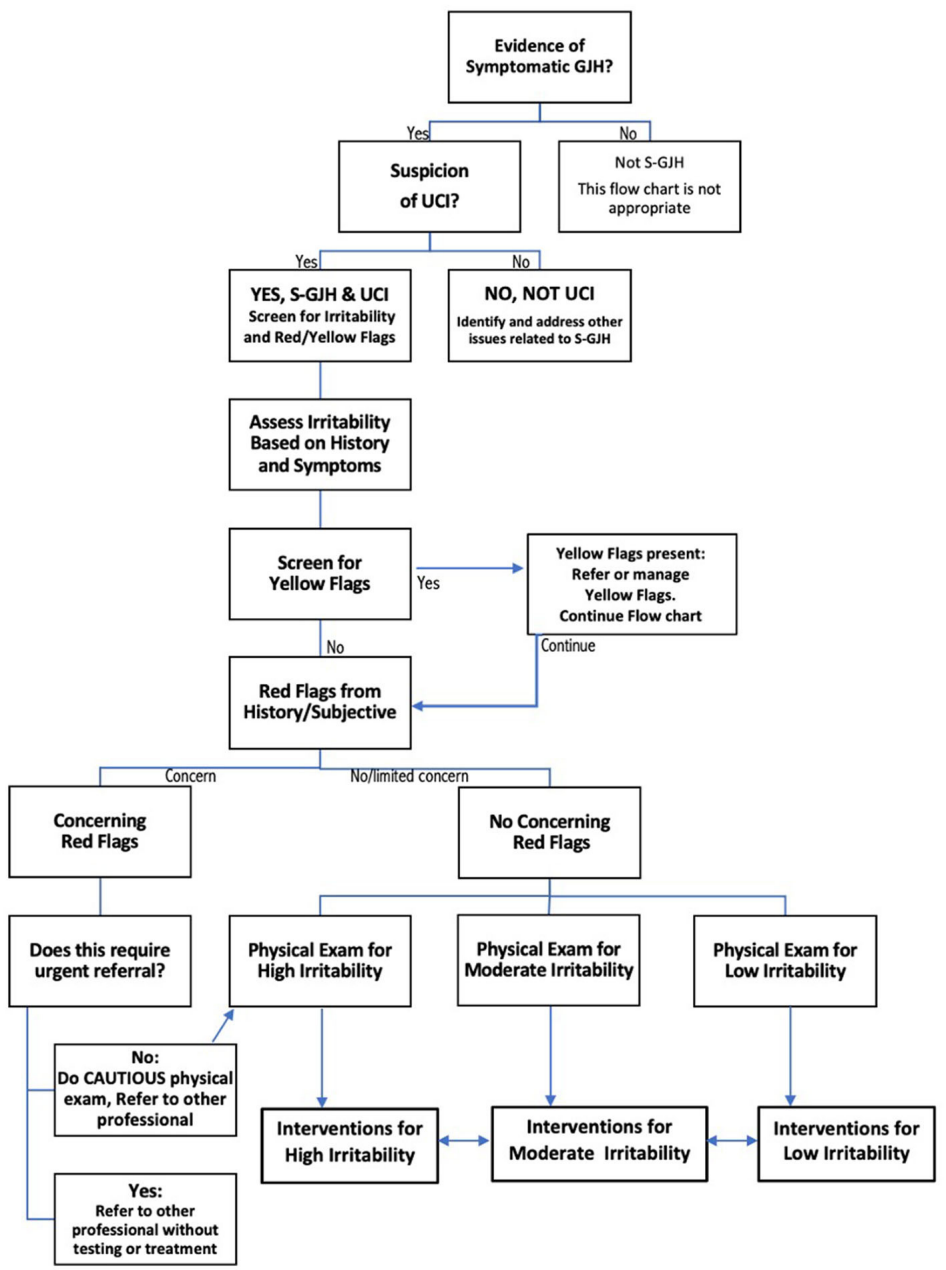
Flow chart describing decision-making for safe screening, physical examination, and intervention in patients with upper cervical instability (UCI) associated with symptomatic generalized joint hypermobility (S-GJH). Bidirectional arrows between High, Moderate, and Low Irritability Interventions reflects the fact that patient status can change, either through recovery or flare. Modified slightly from (3) to create a single flow chart.

Upper cervical instability (UCI) is a potentially disabling complication of hypermobile Ehlers-Danlos Syndrome and Hypermobility Spectrum Disorders (hEDS/HSD) (1, 2). In the absence of evidence-based guidelines regarding physical therapy assessment and management of this population, a recent international expert consensus provided clinical decision-making recommendations for this population. The consensus group recommended ongoing decision-making regarding irritability of the UCI to avoid physical examination tests or interventions that could provoke a flare, as well as screening for Red Flags (RF) and Yellow Flags (YF). Figure 1 shows the decision-making guidelines for screening, physical examination, and intervention in symptomatic patients with UCI (3). The consensus recommendations fill a void in treatment guidance for hypermobility-related UCI. However, it is unclear how these expert recommendations can be applied in practice, and whether they provide a framework for systematic research regarding diagnosis, prognosis or treatment.

Currently, surgical fusion is the only evidence-based treatment for hypermobility-related UCI (4), and that evidence is not yet strong (5, 6). Fusion for craniocervical instability (CCI) and atlantoaxial instability (AAI) are problematic with a high percentage of patients requiring revision surgery, especially if the initial surgery was performed by a non-EDS specialist (7). Surgical success is also affected by patient selection, experience of the surgeon, technique used, and the anatomical variant. Even in expert hands with strict eligibility screening, 25% of fusion patients report no improvement in quality of life (4). Furthermore, all EDS patients have a high risk of surgical complications, including spinal surgery (8). This contributes to the increased healthcare costs for people with HSD/hEDS, which are $11,600–$21,100 more than for similar non-hypermobile people (9). Given limited high quality research regarding effectiveness of surgical management of UCI in this population (5), the limited number of surgeons with the necessary EDS expertise, cost and risks associated with fusion and the number of patients for whom fusion is not appropriate, it is important to explore conservative treatment options.

Joint stability is provided by three components: passive structures such as ligaments, muscles acting on joints, and the nervous system providing feedback and motor control. Although hypermobility occurs when ligaments are lax, instability occurs when the muscles and nervous system are unable to sense and control joint motion. People with hypermobility are known to have compromised proprioception (10) and motor control, and chronic neck pain is associated with further compromises in proprioception and muscle function (11). Neuroplasticity is the process by which the nervous system adapts to change. Maladaptive changes occur in response to injury, while beneficial neuroplasticity occur in response to appropriate rehabilitation. Neuroplasticity requires active, challenging, goal-directed movement with feedback about accuracy (12, 13). Pressure and laser biofeedback training meet the requirements to achieve therapeutic neuroplasticity by providing specific feedback on purposeful active movements, and have been shown to benefit chronic neck pain and instability (14–16). In fact, motor control training of the lumbar spine enhances cervical motor control and decreases neck-related disability, showing the benefit of motor control training remote from the injury (15).

The current brief report describes neurological signs and symptoms for three patients with high-irritability UCI associated with the connective tissue disorder, hEDS/HSD. The report applies the recent clinical decision-making tool for assessment and physical therapy management of these patients, using a multistep process involving screening for Red and Yellow Flags and determining irritability of the condition to determine what physical examination tests and interventions are safe to perform (17). This report also demonstrates how a neuroplasticity approach to managing UCI can be applied to benefit patients with severe UCI and varying outcomes that may occur (14, 16). This treatment approach, described in Appendix A, focuses on 5 components:

1. Posture and alignment awareness training to minimize strain; using supports and braces as needed.
2. Retraining common daily movements such as walking and hinge hip to optimize alignment and motor control.
3. Slow, isometric, or small-range proprioception, stabilization, and movement training, often using biofeedback.
4. Larger and other movement training and strengthening using previously learned proprioception and movement control.
5. Application of improved stabilization and movement control to functional activities.

## 2 Materials and methods

### 2.1 Patient characteristics: history and subjective information

The current study was a retrospective observational design using patient data obtained during the medical history, subjective report, screening and assessment of condition irritability prior to physical examination and intervention (see Figure 1). Data were collected, with patient consent, from medical records and patients fact-checked the data. Interventions were customized for each patient but followed the principles of the neuroplasticity approach.

Because this was a pilot study, patients were purposefully selected. Inclusion criteria were hypermobility, severe UCI, and appropriate for conservative care. Severe involvement ensured that all components of the decision-making tool were utilized. Patients were selected with varied outcomes explore what elements in the decision-making tool might predict prognosis. Another inclusion criterion was complete data, including long-term follow-up. The small sample size was known to be a limitation, but was balanced against the challenges of compiling, organizing, and reporting such complex patient data.

Jay, a 44-year-old male, had seen a local, non-EDS neurosurgeon who concluded that Jay did not have cervical pathology. Jay subsequently saw an EDS neurosurgeon who diagnosed UCI based on upright cervical flexion/extension Magnetic Resonance Imaging (MRI) and recommended 3 months of conservative care before considering surgery. Emma, 40-year-old female, was diagnosed with Chiari malformation, UCI, Postural Orthostatic Tachycardia Syndrome, and hypermobility by an EDS-knowledgeable neurosurgeon after an upright cervical flexion-extension MRI. Kay, a 30-year-old female, was diagnosed with mild UCI and tethered cord by an EDS-knowledgeable neurosurgeon; her UCI symptoms worsened after tethered cord release. Our primary outcomes measures, Neck Disability Index (NDI) and SF-36, are shown in Appendix B. The NDI evaluates the degree of disability in patients with cervical spine injuries (18). The SF-36 is a general health survey that assesses eight health domains (19).

The first step in the clinical decision-making process (Figure 1) is determining that the patients have symptomatic Generalized Joint Hypermobility. Emma had a Beighton score of 7/9, and Kay had a Beighton score of 9/9; both met the requirements for HSD. Jay was not currently hypermobile but reported a history of hypermobility during adolescence, including excessive neck mobility and being “very flexible.” Jay scored 2 out of 5 on the 5-Item Questionnaire (20) and, therefore, had historical hypermobility (17).

Specific subjective and history findings are shown in Table 1. Jay's primary complaints began when he started sitting and using a laptop at the kitchen island 8–10 h/day. He reported additional symptoms, including difficulty walking, ankle instability, coccyx pain, imbalanced ribs and hips, pelvic floor weakness, and feeling “normal” during the upright cervical flexion-extension MRI in prolonged neck extension. Prior to PT, Jay had flared following chiropractic adjustment with an activator to the upper cervical spine, which provoked UCI symptoms, leaving him bed-bound, requiring a soft collar, and unable to stand or walk the following day. Emma reported an insidious onset of symptoms also noted in Table 1, plus symptoms of high heart rate fluctuation, migraines, fatigue, irritability, left-hand tingling, numbness, loss of fine motor control and weakness, photophobia, “shiny vision and moving dark spots,” icepick feeling back of skull. Emma was regularly bed-bound due to symptoms. Kay had mild UCI before starting PT, but her UCI symptoms flared after tethered cord release. In addition to symptoms listed in Table 1, she had migraines and right-side weakness that would come and go, triggered by light, sound, and smell; her symptoms increased with a 2-day trial of rigid cervical collar use. However, tilting her head 1 cm left temporarily resolved all symptoms.

**Table 1:** Suspicion of upper cervical instability symptoms and history.

	C/S^*^	**Jay^†^**	**Em^†^**	**Kay^†^**
**Musculoskeletal UCI**				
Heavy/bobble head, patient feels like they need to support or brace their head to decrease symptoms	S	Y	Y	Y
Apprehension about initiation or maintenance of neck movement or travel in vehicle	S	Y	N	Y
Lump in throat, trouble swallowing	S	Y	Y	Y
Consistent clicking or clunking in the neck associated with neck movement	S	Y	N	Y
Cervical sensorimotor symptoms such as tinnitus, dizziness	S	Y	Y	Y
Sub-occipital headaches	C	Y	Y	Y
Yoke/coat-hanger distribution pain	C	Y	Y	Y
Neck tension, muscle spasm	C	Y	Y	Y
Brain fog	C	Y	Y	Y
Inconsistent or poor response to treatment for the neck	C	Y	Y	Y
Sleep disturbance, snoring, sleep apnea	C	Y	Y	Y
**Neurological UCI**				
Lump in throat, choking, trouble swallowing, voice changes	S	Y	Y	Y
Symptoms of dysautonomia (especially if not responding to standard treatment), persistent anxiety, functional GI dysfunction, poor temperature regulation, heat intolerance, pre-syncope	S/C	Y	Y	Y
“Boat rocking” instability (not due to musculoskeletal issues)	S	Y	Y	Y
Ataxia: Poor coordination (not due to joint instability)	S	Y	N	Y
Facial tingling/numbness	S	Y	N	Y
Pulling sensation in face, head, teeth, tongue (muscle contraction, not just pain)	S	N	N	Y
Vision changes-trouble with convergence, double vision, aura (teichopsia)	S	N	Y	Y
Dystonia: Involuntary muscle contractions causing involuntary movements or postures	S	N	N	Y
Intermittent dysesthesias in the limbs, not associated with local issues	S	N	Y	Y
Sleep disturbance, snoring, sleep apnea	C	Y	Y	Y
*Report of seizure-like activity, diagnosis of “non-epileptic seizures” or “pseudo seizures”^‡^*	S	N	N	Y
*Drop attacks not associated with dysautonomia (e.g., provoked by neck motion, or without dizziness common in POTS) ^‡^*	S	N	N	Y
*Severe or frequent changes in cognitive status^‡^*	S	N	Y	Y
*Rapidly progressing neurological signs with decreasing functional status^‡^*		N	Y	Y
*Increased bowel/bladder control dysfunction^‡^*		N	N	Y
*Headache worse with coughing, sneezing, bowel movement (Valsalva) ^‡^*		N	Y	Y
*Need to use a walker or wheelchair due to moderate or intermittently severe problems with coordination and balance rather than pain or weakness^‡^*		N	Y	Y

^*^S, Highly Suggestive finding for UCI (high specificity based on expert consensus) (3). ^*^C, Common finding in UCI (high sensitivity based on expert consensus) (3). When the consensus article (3) did not classify a test, this has been left blank. ^†^Y, Yes, symptom/history present; N, No, symptom/history not present; NA, Not Assessed. ^‡^Red Flag symptoms and history are listed in italics.

Red Flag (RF) symptoms and history are noted in Table 1 in italics. Jay had no RF symptoms but experienced occasional unsteady gait and a period of bowel urgency. Emma had several RF symptoms, including frequent cognitive changes where she “would be talking and then not present,” balance and coordination issues making it “hard to get her legs to function.” Kay exhibited all the RF inconsistently, so not all the RF were present at time of initial evaluation. Kay demonstrated rapidly progressing RF, including seizure-like activity, drop attacks, severe and frequent cognitive changes, and neurological signs leading to decreased functional status.

Yellow flags (YF), psychosocial factors that may impact intervention, may be assessed using a variety of screening tools. We chose to use the OSPRO-YF (Optimal Screening for Prediction of Referral and Outcome Yellow Flag) (21), which assesses negative mood, fear avoidance, and positive affect/coping that may impact physical therapy. Each of our patients presented with some YF at the start of care. Jay initially had very high anxiety, but we felt it would not interfere with his progress because he had strong family support. Emma had several YF initially due to the stress of being bed-bound and parenting 3 young children. Kay had all possible YF due to poor support systems, the stress of her health issues, living initially with a roommate and later alone, with her parents living over 2 h away. Psychosocial issues were a concern for both Emma and Kay, but PT had limited options to address them.

### 2.2 Assessment of irritability

Assessment of the irritability of the patient's condition is based on three factors: Condition is severe, Condition is easily flared, and prolonged time to calm after flare (3). Detailed assessment of irritability for Jay, Emma, and Kay is shown in Appendix C.

Jay met all three conditions for high irritability, with severe disability (see NDI and SF-36 measures in Appendix B), easily flared, and slow to resolve. For example, he was repeatedly unable to stand or walk for a day following chiropractic adjustments, taking several months of intermittent bed rest to settle to a pre-flare state. Jay also had a history of not tolerating any neck exercises. Emma met all three criteria, experiencing severe flares and prolonged recovery after events like attending a family wedding, necessitating the use of a hard cervical collar and bed rest, and feeling she was back to pretreatment status. Kay met all three criteria with severe flares and prolonged time to calm. For example, before starting PT, Kay had multiple neurological episodes and emergency room visits in response to chiropractic adjustments to her neck.

### 2.3 Physical exam

Figure 1 shows that physical examination tests are selected based on the patient's subjective history of irritability level. Because all three patients had high irritability, we only performed tests “Safe for All Patients” (test results are in Table 2). Tests for patients with moderate or low instability were not considered safe to perform, and those are listed in Appendix D.

**Table 2:** Physical examination testing for upper cervical instability: tests safe for all patients.

**Tests safe for all patients**	XCS^*^	**Jay^†^**	**Em^†^**	**Kay^†^**
**Observation based tests**				
Posture, full body, sitting and/or standing, and segmental alignment	XC	+	+	+
Breathing pattern (chest vs. diaphragmatic, excessive accessory muscle use)	XC	+	+	+
Significant muscle guarding or reluctance to move neck	C	+	+	+
Observe gait for gross and fine motor dyscoordination not due to other joint hypermobility	S	+	+	+
*Observe for cranial nerve VII dysfunction: Lip drooping, unequal smile, eyelid twitching^‡^*	S	-	+	+
*Observe for dystonia, myoclonic jerking^‡^*	S	-	-	+
*Ataxia, gross neurogenic gait abnormalities, inability to perform tandem gait, Romberg sign present^‡^*		+	-	+
*FASTER Indications of stroke: Face, Arms, Stability (standing), Talking, Eyes. R is for React. ^‡^*		-	-	§
**Neurological tests**				
Testing of hand dexterity (need to distinguish from finger hypermobility). E.g., grip release test	S	-	-	-
*Cranial nerve III, IV, V, VI tests: Altered visual field, eye movement, unequal pupil size, amblyopia (lazy eye), facial sensory loss^‡^*	S	-	-	+
*Reflex tests not involving neck: e.g., Hoffmann, Babinski, clonus, hypertonia^‡^*	S	-	+	+
*Cranial nerve X, XII tests: Uvula, tongue (avoid gag), speech or swallowing dysfunction, choking^‡^*	S	+	+	+
*Dysdiadochokinesia: e.g., rapidly alternating pronation/supination, grip release, fast finger or foot tapping^‡^*	S	-	-	-
*Abnormal vertebrobasilar insufficiency tests with auditory and vision changes, evidence of vertigo, presyncope or syncope^‡^*		-	+	+
**Other tests**				
Palpation for muscle spasm, especially suboccipitals, sternocleidomastoid, levator scapulae, upper trapezius	S	+	+	+
Use of a rigid cervical brace for several weeks decreases signs and symptoms	S	¶	+	¶

^*^S, Highly Suggestive finding for upper cervical instability (high specificity based on expert consensus). ^*^C, Common finding in upper cervical instability (high sensitivity based on expert consensus). ^*^X, Contributing Factor. When the consensus article (3) did not classify a test, this has been left blank. ^†^ “+” indicates positive test outcome, “-” indicates negative test outcome. ^‡^Red Flag tests are noted in italics. ^§^Kay did not present with FASTER signs of possible stroke at the initial evaluation but did present with them at multiple future visits; when this occurred, she was referred to the emergency room for assessment. ¶ Jay was worse in a rigid collar, but better in a soft collar. Kay demonstrated increased neurological signs and symptoms in a rigid collar.

Red flag tests, shown in italics in Table 2, include neurological and neurovascular assessments crucial for identifying serious underlying conditions requiring caution and possibly a neurosurgeon referral (see Figure 1). Jay had 2/9 physical test RF, Emma had 4/9, and Kay had 8/9. If multiple concerning RF signs like bulbar symptoms or myelopathy are present, we would normally consider referral to a neurosurgeon; each of our patients had already been evaluated by a neurosurgeon. Patients with moderate or high irritability UCI should be educated to recognize newly developing or worsening RF symptoms requiring urgent medical attention; this process is called “safety netting” (22).

### 2.4 Interventions

**Figure 2:**
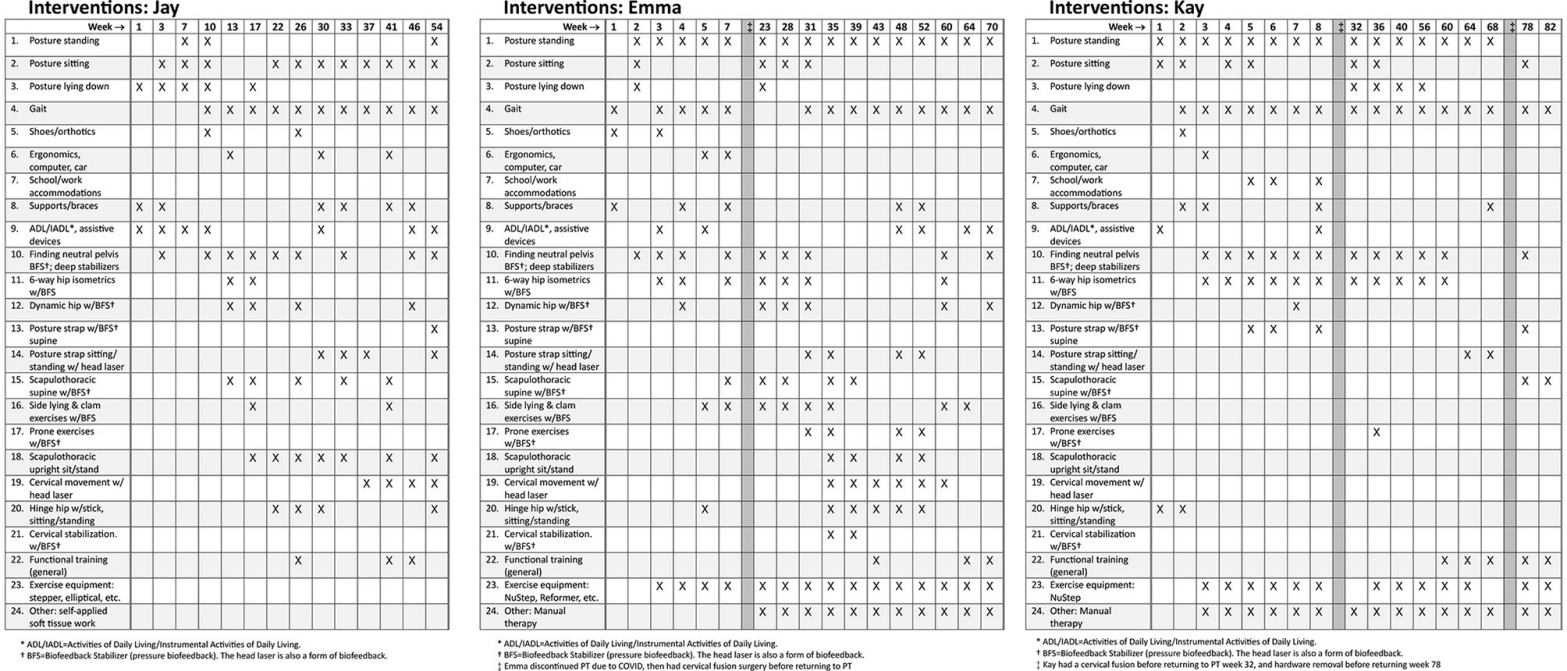
Treatment programs for Jay, Emma, and Kay. This does not accurately reflect treatment frequency and interventions are marked if they were included during that week or surrounding weeks. See Appendix A for more details about each of the interventions in the neuroplasticity training program. Surgeries are noted as dark vertical bars.

Treatment used a neuroplasticity-based proprioception and motor training approach based on “Finding Functional Foundations”™. Specific treatments over time are shown in Figure 2, with brief descriptions of each treatment approach as described briefly in Appendix A. Treatment frequency is shown as week number in Figure 2. Each visit was typically 60 min. Based on high irritability in the subjective and physical exam, the following interventions were deemed unsafe for all three patients: exercises involving neck movements, chin tucks or cervical isometrics, axial loading or distraction, cervical mobilizations or manipulations, and positions that create neural tension or isometric load to the cervical spine (3). Initial treatment focused on HSD/hEDS and UCI education as follows: recognizing signs and symptoms that trigger emergency or urgent follow-up (“safety netting”) (22), self-care in those situations (e.g., wear cervical brace), and functional training for posture and joint protection during essential activities of daily living (ADLs) such as bathing, brushing teeth, brushing hair, washing hair, sleeping postures, eating, and other ADLs. UCI education also included posture and body mechanics training, assessment for orthotics and bracing, body awareness and mindfulness training, pain management and self-care education, activity pacing, breathing, and gentle manual therapy remote from the instability.

Sensory and motor control training included: systematic postural awareness practice; gait training; extensive lumbopelvic, scapulothoracic, and cervical stabilization using pressure biofeedback; cervical proprioception and motor control training using head laser, and full-body exercises integrating stabilization and mobility with pressure or laser biofeedback. All exercises were performed slowly engaging muscles over 5 seconds, maintaining 5 second hold while re-checking alignment, and gradually releasing over 5 seconds using gradually graded muscle effort to emphasize activation of deep stabilizer muscles. Most exercises involved biofeedback. Patients progressed to using these controlled movements during functional activities or exercises, such as hinge hip or using gym equipment. See Appendix A for more detail about the motor control exercises. Regular reassessment ensured treatment plans remained safe, effective, and responsive to the patient's fluctuating symptoms and functional abilities.

After his in-person consultation, Jay had telehealth appointments assisted by his wife, and he worked with a local physical therapist under our guidance for functional exercises and gentle soft tissue work. Jay's first 6 weeks of PT focused on education on safe ADL strategies, body mechanics, using supports, and lumbar stabilization biofeedback training performed in bed due to his inability to get to the floor. Weekly modifications helped manage flares. At 8 weeks, we added lumbar/hip and scapulothoracic stabilization with biofeedback and scapulothoracic and hinge hip at 16 weeks. At 24 weeks, we began functional training using new motor control patterns. Jay was seen once a week for 6 months, decreased to every other week for 18 months, then every month for approximately 8 months for a total of 3 years (see Figure 2).

Emma initially attended PT 1–2 times per week for 6 weeks, focusing on education, ergonomics, ADLs, instrumental ADLs, and lumbar stabilization biofeedback training (see Figure 2). Although she initially progressed well, she stopped PT due to COVID restrictions. After about a month away from PT, severe flares kept her bed-bound 90% of the time, making it difficult to care for her children. Four months after starting PT, she opted for O-C3 fusion. She resumed PT 1–2 times per week post-fusion, focusing on self-care, pain management, postural alignment, soft tissue work, deep tissue laser, and dry needling, gradually reintroducing previous neuroplasticity treatments. She progressed to scapulothoracic biofeedback and stabilization, cervical motor control head laser, cervical biofeedback stabilization exercises, and then to functional training, decreasing PT to once a week. Several surgeries interfered with her progress: removal of loose fusion hardware, ankle surgery, and tethered cord release. Overall, Emma attended PT off-and-on for about 4 years.

Kay began treatment post-tethered cord release, attending PT 1–2 times per week for 2 months, focused on education, postural awareness, alignment, supports, and bracing, ADLs/IADLs, neuroplasticity proprioceptive biofeedback exercises, lumbar/hip stabilization exercises (see Figure 2). Her UCI symptoms gradually worsened, leading to increased neurological episodes and flares with any alignment or resistance exercises. These episodes compromised her work and daily activities. She was referred back to neurosurgery, who performed an O-C3 fusion. After the fusion, she returned to PT 1–2 times per week for nerve calming strategies, joint protection, pain neuroscience education, gentle cervical and cranial manual therapy, and adding light resistance functional exercises when not in a flare. However, she made minimal progress due to frequent, severe seizure-like neurological episodes. Eight months later, her fusion hardware was removed, with no improvement. Kay attended PT for over 2 years, starting and stopping multiple times due to surgeries and high irritable flares, and has continued PT inconsistently over the last 10 months.

## 3 Results

### 3.1 Outcomes

Final NDI, and SF-36 outcomes are reported in Appendix B. Jay's NDI scores improved from complete disability at the start of care to no disability. Jay's SF-36 assessment initially indicated very poor health status in all categories, with significant improvement over time. Jay is currently back to work full-time as an education consultant, occasionally traveling overseas. He has returned to normal function and often does not even think about his cervical instability. He continues with his home exercise program and walks several miles daily. He reports occasional short-duration mild flares that he can manage.

Emma's NDI scores showed complete disability initially, improving to severe disability after several months, then to mild disability following O-C3 fusion and PT. However, her symptoms worsened due to hardware loosening, leading to severe disability again. After hardware removal and a tethered cord release, she improved over the last year to mild disability. Emma's SF-36 scores ranged from 0–100%, indicating poor to good in different health aspects at the beginning of care, with all categories improving from 16%−100%. Her status has fluctuated but she is now able to fulfill responsibilities as a mother and work full-time at a biomedical science lab. She continues with PT for strengthening and manual therapy and uses self-care techniques she learned in PT. She reports occasional mild to moderate flares that vary in intensity and duration.

Kay initially had fairly mild neck problems but developed severe disability post-tethered cord release, worsening over time and requiring O-C3 fusion and subsequent hardware removal. Her NDI scores fluctuated between complete and severe disability. Kay's SF-36 scores also showed significant variability with overall functional decline from the start to end of care. Due to worsening symptoms, Kay could not return to her full-time job as an operating room scrub technician. When she feels well, she currently works part-time as an administrative assistant with a flexible schedule and ergonomic accommodations. She continues to have frequent severe and intense flares that last for a long time. Kay was referred back to neurosurgery, who decided that no further surgical treatment was warranted at this time. Kay continues to consult other specialists to help manage her neurological symptoms and search for answers. She still occasionally attends PT during flares for neural calming techniques and manual therapy.

Outcomes for these patients were quite varied. Jay would no longer be classified as UCI or, at most, would have low irritability UCI. He had no yellow or red flags after PT. Emma would be classified as low-moderate irritability UCI with few yellow and no red flags. Kay would be classified as moderate-high irritability UCI and continued to have yellow and intermittent red flags.

## 4 Discussion

This brief report describes the complex neurological presentation of patients with hEDS/HSD resulting in UCI. Although this connective tissue is most often associated with joint problems, the current report demonstrates neurological presentation that is increasingly recognized among these patients (1, 2). It also demonstrates how the expert consensus guidelines (3) can help clinicians screen, assess, and manage hypermobile patients with UCI safely. By determining that these patients had highly irritable UCI based on history and subjective findings, we were able to determine which physical examination tests would avoid flares during the physical exam. These patients also show how RF and YF can alert providers to factors that may impact outcomes. The presence of RF indicates greater irritability of the nervous system, which suggests greater instability and increased risk of flare due to inappropriate testing or treatment (23).

A position statement regarding examination of the neck for vascular pathology prior to initiating PT also noted the importance of identifying subjective and physical RF suggesting cervical instability (22). A guideline for identifying serious spinal pathology also recommends screening for RF during clinical decision-making (24). The current brief report builds on prior reports suggesting grading scales rating the level of risk associated cervical vascular pathology (22), concussion (23), and spinal pathology (24). The grading scale for concussion (23) used “symptom instability” to rate irritability and severity post-concussion, showing the importance of assessing irritability in these neurological conditions. Evidence suggests that RF are most useful for clinical decision-making, but might not be associated with prognosis (24). Further research could determine whether the presence or number of RF predicts prognosis.

The results suggest that the expert consensus guidelines (3) might be helpful for predicting outcomes in patients with UCI. For example, Jay had 0/7 subjective and 2/9 physical examination RF; he progressed well with conservative care and did not need fusion. Emma had 4/7 subjective and 4/9 physical RF; although she was improving with PT, she opted for fusion because COVID restrictions limited PT access, difficulty with managing flares, and parenting demands. Kay had 7/7 subjective and 7/9 physical RF; she continued to have severe and prolonged seizure-like neurological episodes and flares that are unresolved despite fusion and conservative care. Future research might build on the current work to develop a validated scoring system for RF in UCI to identify patients at highest risk for poor outcomes.

Psychosocial factors reflected as yellow flags (YF) may have contributed to outcomes. Jay had excellent social support and a better outcome. Emma, whose initial physical presentation was only slightly more severe than Jay's, had multiple psychosocial stressors and a poorer outcome. Kay's continued difficulties might have lessened if she had more support to manage psychosocial stressors. These results are consistent with research showing poorer PT outcomes in patients with multiple YF (21), and the ability for YF to improve prediction of outcomes in 12 months (25).

Although there are publications about radiological diagnosis and surgical management of UCI in HSD (4, 6, 7, 26), we believe this is the first report describing conservative care for hypermobile patients with severe neurological involvement due to UCI. Several case reports describe conservative care for UCI in patients with rheumatoid arthritis (27) or whiplash, but those patients had less severe involvement and did not have neurological findings or RF (28).

Neuroplasticity is essential for improving functional outcomes in patients, as it underlies the brain's ability to reorganize and adapt in response to therapeutic interventions. Specific approaches, such as task-specific training, utilize use-dependent motor learning to induce structural changes in the brain, promoting long-term improvements in motor control and efficiency. Instructive motor learning, where patients receive feedback to correct movement strategies, engages cognitive processes to facilitate quick adaptations in motor behavior, including motor control and stability. Future research should focus on identifying which combinations of motor learning mechanisms most effectively drive neuroplastic changes and lead to sustained improvements in functional abilities, particularly in hypermobile individuals (12, 13).

This report also demonstrates how a neuroplasticity-based treatment approach can safely manage patients with UCI with varying degrees of success. This treatment approach is based on principles of neuroplasticity, so each patient progresses at his or her own pace. Patients with mild or moderate UCI would engage all the same components, but would likely progress more quickly and have fewer setbacks. Lumbar (15), and cervical pressure biofeedback (16) have been shown to be effective in patients with chronic neck pain but have not yet been studied in hypermobility-related UCI. Similarly, laser biofeedback has demonstrated effectiveness for chronic neck pain (14) but has not yet been assessed in UCI. The patients also show that cervical fusion is not always an instant solution, and PT may still be beneficial in addressing risk factors that contribute to cervical instability. Hopefully, this neuroplasticity approach can be assessed in future experimental research.

A strength of this brief report is that it provides a detailed description of the complex neurological presentation and physical therapy management for people with severe UCI using a methodical approach supported by expert consensus. The small sample size limits generalizability to a larger patient population, and purposeful sampling means that we cannot say these are typical responses. Retrospective design limits our ability to show causation. Although all were treated using a neuroplasticity approach, the variability of specific treatments used for each patient limits conclusions about any specific component of treatment. Two of the patients ultimately had cervical fusion but returned to PT afterward to recover strength and function and manage symptoms. There was significant variability in how often and how long each patient was seen in PT. Furthermore, these patients were seen in PT over extended time periods, ranging from 2–4 years of sometimes intermittent PT. The duration of PT care described in this report is both a strength and limitation. It is a strength because we have long-term follow-up, but it is a weakness because most patients do not have access to such extensive PT care. Because these patients all presented with severe instability, it is likely that patients with mild or moderate instability could be successfully treated in fewer PT sessions.

This report provides a first step toward systematic research. The expert consensus provides a viable framework for assessing patients, stratifying intervention by severity, and establishing prognosis. However, several challenges for prospective clinical research are clear. These patients have frequent flares of UCI or other hypermobility-related problems that lead to surgery or setbacks. It would be challenging to fully standardize an intervention for this population because tolerance to treatment is so variable. Also, the interventions here lasted up to 82 weeks, which would be difficult to implement for larger samples. Clinical studies might benefit from starting with patients who have moderate UCI rather than severe, as used here.

In conclusion, this case series provides a detailed description of potential neurological presentation in patients with UCI due to the connective tissue disorder hEDS/HSD. These cases demonstrate how recent expert consensus recommendations can be used to safely evaluate and manage patients with severe and irritable UCI. The neuroplasticity-based treatment approach described here is consistent with the consensus recommendations. This report also illustrates some of the challenges in treating these patients, including frequent flares, related or unrelated surgeries or medical issues, and slow and inconsistent response to conservative treatment. The current work, therefore, serves as a pilot study to guide future research. Future research should validate both the UCI clinical decision-making recommendations and conservative treatment approaches for patients with hEDS/HSD and UCI.

## Data Availability

The datasets presented in this article are not readily available because of ethical and privacy restrictions. Requests to access the datasets should be directed to the corresponding author.
