# Supplementary material for: Presentation and physical therapy management using a neuroplasticity approach for patients with hypermobility-related upper cervical instability: a brief report
Source: Front Neurol. 2024 Nov 8;15:1459115. doi: 10.3389/fneur.2024.1459115 (PMC11581960; doi:10.3389/fneur.2024.1459115)
Supplement: Supplementary file 1 [file Data_Sheet_1.pdf]

Supplementary Material

Presentation and physical therapy management using a neuroplasticity approach for patients with hypermobility-related upper cervical instability: A brief research report

Susan Chalela, Leslie Russek

Appendices

- Appendix A. Brief description of neuroplasticity interventions based on “Finding Functional Foundations”™ (FFF) approach.*
- Appendix B. Patient Outcome Measures (Initial and Follow-Up).
- Appendix C. Determining the Irritability of Upper Cervical Instability.
- Appendix D. Physical Examination Testing for Upper Cervical Instability: Tests for Moderate and Low Irritability Patients Only.

Appendix A.

Brief description of neuroplasticity interventions based on “Finding Functional Foundations”™ (FFF) approach.

Posture standing: “5-Block Standing” posture instructs patients to stand with 1) weight evenly balanced on the feet, 2) knees slightly unlocked, 3) pelvis neutral, 4) shoulders centered and shoulder blades back and down, 5) head balanced over shoulders. Alignment is maintained through proper balance rather than excessive muscle effort.
Posture sitting: “5-Block Sitting” posture instructs patients to sit with 1) weight evenly balanced on the feet, 2) knees aligned with hips and heels, 3) pelvis neutral, 4) shoulders centered and shoulder blades back and down, 5) head balanced over shoulders. Alignment is maintained through proper balance rather than excessive muscle effort.
Posture lying down: Proper alignment supported with pillows supporting the head and all limbs, as needed.
Gait: 1) Feet do not cross boundary line, 2) soft knees with no hyperextension, 3) heel strike centered and soft, 4) push-off evenly from front of foot, 5) neutral pelvis and upper body alignment as for “5-Block Standing”
Shoes/orthotics: As needed to properly align the lower extremities and trunk in standing.
Ergonomics, computer, car: Optimal body alignment at the computer and car, using supports for back and arms, as needed.
School/work accommodations: Modification of school or work demands to meet the tolerance of patients with HSD/hEDS.
Supports/braces: Supports and braces to assist with proper body alignment and movement in sitting, standing, walking.
ADL/IADL, assistive devices: Addressing the patient’s ADL and IADL challenges through body mechanics training and assistive devices.
Finding neutral pelvis w/BFS; deep stabilizers: Hook-lying exercise with BFS to find and maintain neutral pelvis. Self-assessment of body alignment using “5-Point Alignment Check”: 1) feet balanced and weight equal, 2) knees hip width apart, 3) belly breathing, 4) ribcage (breastbone) still (not chest breathing), 5) shoulders centered and shoulder blades back and down.

6-way hip isometrics w/BFS: A set of 6 isometric hip/core exercises done in hook-lying, using the BFS to ensure that neutral pelvis is maintained. Emphasis is on slow, controlled muscle activation and body awareness. Isometrics include hip adduction, abduction, internal rotation, external rotation, flexion and extension while maintaining neutral pelvis using the BFS. All exercises begin with activation of the deep stabilizing muscles.
Dynamic hip w/BFS: A set of dynamic hip/core exercises done in hook-lying, using the BFS to ensure that neutral pelvis is maintained during .slow, controlled, small range motion. The dynamic movements include knee fall-outs, heel slide, and foot lifts maintaining neutral pelvis using the BFS. All exercises begin with activation of the deep stabilizing muscles.
Posture strap w/ BFS supine: Using a 10-foot yoga strap wrapped around the shoulders and upper back with gentle tension that assists proper scapulothoracic alignment while maintaining neutral pelvis using the BFS.
Posture strap sitting/standing with head laser: Using the 10-foot yoga strap wrapped around the shoulders and upper back with gentle tension that assists proper scapulothoracic alignment while maintaining neutral pelvis in sitting or standing, using a head-mounted laser for biofeedback to ensure stability of the head and neck. "Target practice" is a process by which the patient centers the laser while in ideal alignment, then closes his eyes while adjusting alignment using "5-Block Sitting" or "5-Block Standing" principles, then opens the eyes and realigns the laser with the target. This is repeated until the laser no longer needs to be realigned after the eyes-closed "5-Block Sitting" or "5-Block Standing" process. Can be done with or without the posture strap.
Scapulothoracic supine w/BFS: A set of scapulothoracic stabilization exercises in hook-lying, using the BFS to maintain neutral pelvis during .slow, controlled, small range motion. All exercises begin with activation of the deep stabilizing muscles. The exercises include shoulder external rotation, shoulder external rotation with shoulder flexion, shoulder external rotation with shoulder abduction, and shoulder external rotation with shoulder diagonal movement.
Sidelying & Clam exercises w/BFS: Side lying with the BFS under the waist, performing a variety of clamshell exercises maintaining lumber stability.
Prone exercises w/BFS: A set of prone exercises with BFS under the stomach, doing small motions lifting either arms, legs, or unweighting the head while maintaining steady BFS pressure.
Scapulothoracic upright sit/stand: A set of scapulothoracic stabilization exercises in standing or sitting, applying body-awareness learned from the BFS to maintain 5-Block Standing or Sitting alignment while doing slow, controlled, small range motion. Exercises include standing wall plank lower and lift and seated row and pulldown.
Arm/leg movement w/ head laser: Biofeedback exercises done in "5-Block Sitting" with head laser. Maintaining stable head laser position while slowly moving an arm or leg.
Cervical movement w/ head laser: Biofeedback exercises done in "5-Block Sitting" with head laser. Keeping the laser along a line while doing small (5º) head motions into rotation or flexion/extension.
Hinge hip w/stick, sitting/standing: strapping a stick to the back and maintaining contact at the head, upper back and sacrum to provide proprioceptive feedback, learning to flex the hip while maintaining spinal alignment. Also sitting on large exercise ball, forward and backward.
Cervical stab. w/BFS: Supine hook-lying with BFS under the cervical spine. Sometimes a second BFS is placed under the lumber spine so patients can monitor both. Activating the deep neck stabilizers and scapulothoracic stabilizers to maintain constant pressure under the cervical spine while doing light cervical isometrics in neutral and small deviations from neutral. Some patients progress to small range dynamic motions maintaining constant pressure in the cervical (and lumbar) spine.
Functional training (general): Functional movements such as stepping over obstacles using hinge hip. Wall slides, without and with gym ball.
Equipment: Training patients to use exercise equipment such as the recumbent bike, rower, stepper, Pilate Reformer or elliptical while maintaining lumbar and cervical stabilization. The head-laser may be used as feedback for stabilization on most exercise equipment.

Abbreviations:
HSD/hEDS = Hypermobility Spectrum Disorder or hypermobile Ehlers-Danlos Syndrome.
ADL/IADL = Activities of Daily Living/Instrumental Activities of Daily Living
BFS = Pressure biofeedback stabilizer

Appendix B. Patient Outcome Measures (Initial and Follow-Up).

	Jay			Em			Kay		
	Initial	Final	**Change**	Initial	Final	**Change**	Initial	Final	**Change**
Neck Disability Index (%)*	90	12	**-78**	84	28	**-56**	50	60	**+10**
SF-36 Physical function (%)†	0	100	**100**	30	60	**30**	80	0	**-80**
SF-36 Emotional role limitation (%)†	0	100	**100**	100	100	**0**	66.7	0	**-66.7**
SF-36 Energy/fatigue (%)†	5	70	**65**	0	35	**35**	40	20	**-20**
SF-36 Emotional well-being (%)†	8	92	**84**	68	84	**16**	44	40	**-4**
SF-36 Social function (%)†	0	100	**100**	25	62.5	**37.5**	37.5	25	**-12.5**
SF-36 Pain (%)†	0	90	**90**	10	35	**25**	32.5	22.5	**-10**
SF-36 General health (%)†	5	85	**80**	20	45	**25**	25	15	**-10**
SF-36 Health change (%)†	0	75	**75**	0	100	**100**	25	0	**-25**

*Neck Disability Index scores disability, so lower numbers are better.

†SF-36 scores ability, so higher numbers are better.

Appendix C.

Determining the Irritability of Upper Cervical Instability

	Jay	Em	Kay
**A. Condition is severe:**			
• Poor tolerance to any time vertical	Y	Y	Y
• Bed bound due to cervical symptoms	Y	Y	Y
• Need to use a walker or wheelchair due to moderate or intermittently severe problems with coordination and balance rather than pain or weakness, or restricted to bed due to cervical symptoms	N	Y	Y
• Extreme cervical spine guarding with fear of movement secondary to severe reactivity	Y	N	Y
• Choking, trouble swallowing, and voice changes	Y	Y	Y
• Profound visual disturbances	N	Y	Y
• Severe nausea with any neck movement	N	N	N
• Functional outcome measure relevant to UCI classified as Severe	Y	Y	Y
**B. Condition is easily flared:**			
• UCI flares are disproportionate compared to provoking insult or activity. e.g., aggravated by minor rapid/unexpected movements/perturbations, traveling in car/bus, prolonged postures.	Y	Y	Y
• Presyncope, syncope, drop attacks or seizure-like episodes with neck extension or rotation.	N	N	Y
• History of excessive provocation associated with previous conservative care including hands-on manual therapy or exercise.	Y	Y	Y
**C. Prolonged time to calm after flare:**			
• Provoked UCI symptoms take excessive time to settle to pre-flare state: e.g., more than 24 h for pain or more than several hours for neurological symptoms	Y	Y	Y
• Pt regularly needs to resort to wearing a cervical collar or bedrest to ease symptoms after a flare	Y	Y	Y
• Inability to tolerate being upright for > 24 h after flare	Y	Y	Y
Irritability Grade			

† Y=Yes, symptom/history present; N=No, symptom/history not present; NA=Not Assessed.

Grading mechanical irritability

Low irritability:

- A, B, and C are all typically absent, or
- B or C might be occasionally present at a low level.

Moderate irritability:

- A, B, or C are intermittently present, or
- A or B or C is frequently present, but not all three consistently.

High irritability:

- A, B, and C are all frequently present.

Modified slightly from Russek, 2022

Appendix C

Physical Examination Testing for Upper Cervical Instability: Tests for Moderate and Low Irritability Patients Only

Tests for Moderate and Low Irritability Patients Only				
	XCS	Jay	Em	Kay
**Other motion and control**				
Thoracic range of motion, range, and quality	XC	NA	NA	NA
Scapular muscle strength and motor control	XC	NA	NA	NA
Excessive use of temporomandibular muscles to provide cervical stabilization (secondary finding)	C	NA	NA	NA
**Neck motion and control**				
Cervical range of motion: Overall, looking for apprehension, range, and quality	CS	NA	NA	NA
Deep neck flexor recruitment efficiency	XC	NA	NA	NA
Cervical stabilizer motor control inhibition and inefficient recruitment (e.g., craniocervical flexion test, suboccipital extensor test)	XC	NA	NA	NA
Sensorimotor tests: Eye-head coordination, trunk-head coordination, smooth pursuit visual tracking	XC	NA	NA	NA
Cervical proprioception: Joint position error	XC	NA	NA	NA
**Other tests**				
Neurodynamic tests may be cautiously performed, eliminating or caution with neck motion	XC	NA	NA	NA
Orthostatic intolerance: NASA lean test or stand test	CS	NA	NA	NA
**Structural tests**				
Cervical axial load in supine	S	NA	NA	NA
Alignment of C1 (manual assessment)	S	NA	NA	NA

* S=Highly Suggestive finding for upper cervical instability (high specificity based on expert consensus)
* C=Common finding in upper cervical instability (high sensitivity based on expert consensus)
* X= Contributing Factor
Table based on Russek, 2022
